# The Finances of the Voluntary Hospitals

**Published:** 1920-07-10

**Authors:** Napier Burnett


### THE FINANCES OF THE VOLUNTARY HOSPITALS.

### By SIR NAPIER BURNETT, K.B.E.

**The following statement, addressed to Sir Arthur Stanley, G.B.E., Chairman of the Joint Council, has been sent to us by Sir Napier Burnett for publication. It contains data on a much wider basis than that included in his recently published Hospital Survey, namely, the financial position of the Voluntary Hospitals throughout Great Britain and Ireland for a period of five years. The statement published previously (see "The Hospital," May 29, p. 236) was limited to England and for the year 1919. In order to avoid overlapping Sir Napier has again purposely excluded from the communication all reference to the London group of hospitals, as they come directly under the purview of King Edward's Hospital Fund.**

It may be taken as a general statement that, up to the end of the year 1914, there was no great hospital problem from the point of view of finance: what problem there was had reference mainly to the increasing need of hospital accommodation. The present investigation has been undertaken with the object of ascertaining to what extent hospital finance has been affected during the years of war, and the following figures relate to the *years* 1915 *to* 1919 *inclusive*.

The inquiry was limited to the three main questions, viz.:

(*a*) The total amount of ordinary income—excluding legacies—received during each of the five years.

(*b*) The total value of legacies—not earmarked for any specific purpose—received during the same periods.

(*c*) The total amount of ordinary expenditure for each year.

The hospitals were specially asked not to include as income any legacies that were earmarked for any special purpose—*e.g.*, endowment of beds, etc., Neither were they to include as ordinary expenditure moneys spent on extensions, new buildings, or alterations, etc., which might be described as extraordinary expenditure.

THE POSITION UP TO DECEMBER 31, 1919.

With these reservations, the following figures, taken from audited hospital accounts, may be accepted as a fairly accurate statement of the financial position up to December 31, 1919.

SUMMARY FOR ENGLAND, WALES, SCOTLAND, AND IRELAND.

Out of a total of some 900 hospitals replies were received from 702; 77 per cent. of the whole.

Of these 702 hospitals 32 stated that they had incurred no deficits and required no special assistance.

The following (Table I. *a*) gives the total of the remaining 670 hospitals:—

TABLE I. *a*.

| Number of Hospitals | Total Ordinary Income excluding Legacies | Total Legacies not earmarked | Total Ordinary Expenditure |
|---|---|---|---|
| 670 | £15,771,346 | £2,061,830 | £17,462,299 |

Income plus legacies ... ... £17,833,176

Expenditure ... ... ... ... £17,462,299

**Excess of Income, plus Legacies, over Expenditure, £370,877.**

The following (Table I. *b*) shows a division of the hospitals into two groups, viz.:—

(1) HOSPITALS SHOWING EXCESS OF INCOME PLUS LEGACIES OVER EXPENDITURE:—

| Number of Hospitals | Total Ordinary Income excluding Legacies | Total Legacies not earmarked | Total Ordinary Expenditure |
|---|---|---|---|
| 259<br>38·65% | £4,250,466 | £324,259 | £3,979,173 |

**Excess of Income, plus Legacies, over Expenditure, £595,552.**

(2) HOSPITALS SHOWING EXCESS OF EXPENDITURE OVER INCOME AND LEGACIES:—

| Number of Hospitals | Total Ordinary Income excluding Legacies | Total Legacies not earmarked | Total Ordinary Expenditure |
|---|---|---|---|
| 411<br>61·34% | £11,520,880 | £1,737,571 | £13,483,126 |

**Excess of Expenditure over Income and Legacies, £224,675.**

Summary showing the financial position of 670 hospitals through Great Britain and Ireland (excluding London) during the five years 1915-1919 inclusive:—

| Year. | Total Ordinary Income excluding Legacies. | Legacies not earmarked for special purposes. | Total Ordinary Expenditure. | Excess of income *plus* free Legacies over Expenditure. | Excess of Expenditure over Income *plus* free Legacies |
|---|---|---|---|---|---|
| | £ | £ | £ | £ | £ |
| 1915 ... | 2,395 348 | 337,738 | 2 561,764 | 171,322 | — |
| 1916 ... | 2,732,865 | 405,983 | 2 960,470 | 178,378 | — |
| 1917 ... | 3,206,788 | 482,452 | 3,460,488 | 228,752 | — |
| 1918 ... | 3,706,837 | 424,747 | 3,986,284 | 145,300 | — |
| 1919 ... | 3,729,508 | 410,910 | 4,493,293 | — | 352,875 |
| Totals ... | 15,771,346 | 2,061,830 | 17,462,299 | 723,752 | 352,875 |

COMMENTS ON THE FIGURES.

While there were individual hospitals which, during the first four years, showed deficits, owing, no doubt, to local conditions, yet, *considered as a whole*, the voluntary hospitals were in a satisfactory financial state up to the end of 1918, as shown above.

During 1919 it will be observed that while expenditure rose by half a million, largely owing to increase in wages, etc., and to number of staff, with their shorter hours, yet the income appears to have ceased expansion, due, no doubt, to the withdrawal of the military capitation grants, and to the effects of increased taxation.

From the above figures you will observe the important part played by legacies in the income of the hospitals. I have included them with the ordinary income, as they are free and unconditional for the hospitals to deal with as they care to; in other words, the hospitals, taken as a whole, have received during these five years more money than was required to meet their ordinary expenditure.

Many hospitals have a fixed rule of treating all legacies, whether free or specially earmarked, as capital, and such a rule may be regarded as sound in principle; but I submit that there are occasions when such a rule may have to be departed from, as, for instance, when a hospital finds its ordinary expenditure to be in excess of income for any year, it would be sound business for that hospital to

**Finances of Voluntary Hospitals—(*continued*).**
make use of the unearmarked money it has received, either as a donation or legacy, which is merely a difference in name, to meet its liabilities.

But many hospitals have not acted in such manner, preferring instead to adhere to their old fixed rule of placing all legacies to capital and overdrawing their credit at the bank in order to meet their deficits. The investment of such capital yields at the most something like 5 per cent., while their bank ovedraft will cost them in these days 6½ to 7 per cent.

Some Expenditure Five Years in Arrear.

One other point calls for special note—namely, extraordinary expenditure, incurred on extensions, additions, or alterations, etc. Some hospitals to-day show a deficit owing to such expenditure, but the great majority of the hospitals confess in their letters to me that, anticipating a slump in prices at the end of the war, all necessary repairs, cleaning, and alterations were postponed, and are now five years in arrears, and ask for assistance towards this end.

Included as ordinary income are the amounts received by the hospitals as capitation grants for the treatment of military patients.

It is true this source of income has ceased, but many hospitals to-day are in receipt of capitation grants from the Ministry of Pensions, while others are replacing the military grants by instituting accommodation for paying patients, and others are receiving a daily levy from their patients towards cost of maintenance; in short, many hospitals are now busily engaged in attempting to secure fresh sources of income. The year 1919 brought a fresh anxiety to hospital authorities in the shape of the necessity for additional accommodation for nursing staff consequent on more nurses having to be employed to meet the demand for shorter hours of service. This is a problem of capital expenditure which, although outside the scope of my present survey, is a matter of very urgent importance.

Conclusions.

From the tabular statements set out above, I suggest for your consideration the following conclusions:

(1) The figures shown as a summary for the country indicate that **the voluntary hospitals, taken as a whole, are solvent!** Their capital in the form of investments, not including the value of buildings, is **considerably over twelve million pounds.**

(2) Table I.*b* shows an important division of the hospitals into

(*a*) those showing an *excess of income plus legacies* of £595,552, and

(*b*) those showing an *excess of expenditure* over income and legacies of £224,675.

But it would, of course, be ridiculous to suggest that the surplus of the one group should be used to wipe out the deficit of the other.

I would particularly direct your attention to the importance of establishing a proper basis for the allocation of any money grants which your Joint Council may be asked to disburse.

Merely to make grants to hospitals showing deficits would be wrong, as in some cases inefficient hospital administration might thus be subsidised, while, on the other hand, to withhold assistance from those hospitals showing no deficit might be penalising good management. The only sound basis for the allocation of grants to hospitals is on the report of inspection of the individual hospital by some authority possessing the necessary qualifications. It is not merely enough to rely on statistical returns, valuable as they may be, as they are not infrequently misleading. So far, there is no finality in hospital administration, and as a science it exists in the hospitals in this country to-day in widely varying standards. I suggest that any council or fund in the position of distributing grants to hospitals ought to exercise its power to the utmost in seeking to raise the standard of hospital administration in this country.

(3) The above-shown deficit for the provincial hospitals amounts to a quarter of a million, and I understand the deficit on the London group amounts to a similar sum.

I estimate that, in order to meet the cost of repairs to buildings, alterations, cleaning, etc., which are now *five years in arrears*, the amount required for both London and the rest of the country would, approximately, be three-quarters of a million.

Owing to the heavy depreciation in the value of invested stock, hospitals would suffer considerable financial loss if they were forced to realise their investments at present, and such action would, by loss of dividends, further lower **the ordinary income.**

One Million Pounds Required.

I suggest that, just as the King's Fund has already agreed to find this sum of a quarter of a million to liquidate the deficit on the hospitals of London, so if a further sum of one million pounds could be obtained to clear off the deficit of all other hospitals, and also to enable all the voluntary hospitals of the country to bring their overdue repairs, alterations, cleaning, etc., up to date, it would thus be possible to restore the hospitals of Great Britain and Ireland *to the position they occupied in* 1914, with this difference: that an increased **income must be** obtained to meet the increased cost of salaries and commodities.

If we are to arrive at a true estimate of the hospital situation in the country to-day, it appears to me to be essential to survey the existing organisation in a comprehensive manner; all the more so before any new scheme of hospital service is decided upon.

So far, only one aspect of the case has been dealt with —namely, the financial position.

Further Matters to be Investigated.

The remaining aspects of the problem to be investigated are:

(*a*) The existing bed accommodation.
(*b*) The additional hospital provision that is required.
(*c*) The sites for any new hospitals.

From my investigations I find that in many localities a considerable extension of the present hospital accommodation is urgently needed, both for patients and for staff; and how these extra beds are to be provided is a matter requiring careful consideration. No doubt much of the pressure on the present available accommodation might be relieved by making use of the beds in some of the Poor-Law infirmaries.

This raises the whole question as to how far the State might be called upon to assist in the direction of providing the new accommodation. On another occasion I hope you will allow me to put before you some suggestions as to how far the State should be called upon to render assistance, and what the basis of such assistance should be.

In conclusion, I would plead for a broader view of this hospital question than that which is generally taken. It is not enough merely to consider the needs of one individual hospital—or even of several hospitals. What we ought rather to seek after is the welfare of the sick and suffering throughout the country, and the provision of adequate hospital facilities for their relief and restoration.

